# Utilizing bedside VExUS and the S-prime velocity for real-time assessment of venous congestion on right ventricular function in heart failure

**DOI:** 10.1186/s43044-026-00788-7

**Published:** 2026-10-06

**Authors:** Pramukh Hegde, Malvika P. Prasad, Kingshuk Saha, Vimal Bhardwaj, Vikneswaran Gunaseelan, Nicolas Orozco, Arjun Alva

**Affiliations:** 1 Critical Care Services, Narayana Health, Bengaluru, India; 2 Centro de Investigaciones Clínicas, Fundación Valle del Lili, Santiago de Cali, Colombia

**Keywords:** S-prime, VExUS, Venous congestion, Heart failure

## Abstract

**Background:**

Right ventricular (RV) function is a major determinant of outcomes in heart failure (HF), yet RV systolic performance and venous congestion (VC) are often evaluated separately. We investigated the relationship between tricuspid annular systolic velocity (S′), a tissue Doppler marker of RV systolic function and systemic VC measured by the Venous Excess Ultrasound (VExUS) score in patients with HF. In addition, RV–pulmonary artery (RV–PA) coupling parameters including TAPSE/PASP and S′/PASP were evaluated across VExUS grades.

**Methods:**

In this prospective, single-center observational study, 155 patients admitted with decompensated HF underwent standardized echocardiography, including VExUS assessment and S′ measurement. Lung Ultrasound Score (LUS), RV–PA coupling indices, clinical characteristics, in-hospital mortality, and acute kidney injury (AKI) were also recorded.

**Results:**

The mean age was 57.5 years, with 68% of the cohort being males. A significant inverse correlation was observed between VExUS grade and S′ velocity (*p* < 0.001). Patients with VExUS Grade 0 had higher S′ velocities (median = 11.0 cm/s), while those with Grade 3 had the lowest (8.5 cm/s). Increasing VExUS grades were also associated with progressive worsening of TAPSE, TAPSE/PASP, and S′/PASP ratios, suggesting impaired RV–PA coupling with increasing venous congestion. Both higher VExUS grades and reduced S′ were associated with more severe pulmonary congestion on LUS (*p* < 0.001 and *p* = 0.003, respectively). Although patients with higher VExUS grades and lower S′ values trended towards worse outcomes, neither parameter significantly predicted in-hospital mortality or AKI.

**Conclusion:**

Systemic VC and RV systolic function are strongly and inversely related in HF. The combined use of VExUS and S′ provides an integrated bedside assessment of hemodynamic status that may help guide decongestive therapy. Integration of RV–PA coupling parameters may further enhance bedside evaluation of hemodynamic compromise in HF patients. Additionally, S′/PASP and TAPSE/PASP demonstrated comparable trends across all HF groups, suggesting that S′/PASP may serve as a practical alternative marker of RV–PA coupling in heart failure patients. Larger studies are needed to determine their prognostic utility for predicting mortality and organ injury.

**Trial registration:**

This trial was registered with Clinical Trial Registry-India (https://www.ctri.nic.in/), Trial Number - CTRI/2024/10/075219 on 14/10/2024.

**Supplementary Information:**

The online version contains supplementary material available at https://doi.org/10.1186/s43044-026-00788-7.

## Background

The right ventricle (RV), long overshadowed by the left, is now firmly recognized as a critical determinant of outcomes in a wide range of cardiovascular diseases. Increasing evidence highlights that RV dysfunction is a major predictor of morbidity and mortality across the HF spectrum [1]. Given its unique crescent shape, thin wall, and sensitivity to afterload, it is particularly vulnerable to pressure and volume overload. RV dysfunction contributes to reduced forward flow, elevated venous pressures, and progressive systemic congestion, ultimately leading to multi-organ dysfunction [2–4].

Tricuspid Annular Plane Systolic Excursion (TAPSE) and tricuspid annular systolic velocity (S’), obtained from the apical 4-chamber view, are widely used echocardiographic measures of RV systolic function [5, 6]. They quantify the longitudinal motion of the RV base toward the apex, offering reliable, accessible indicators of RV performance in routine clinical practice.

While TAPSE is widely validated, S′ offers certain practical advantages, like ability to provide real-time, quantitative assessment which can be easily done at the bedside and has established prognostic value across multiple cardiovascular diseases [7, 8]. It is also noted to have lower incidence of inter-observer variability [9]. Importantly, S′ has demonstrated good correlation with RV ejection fraction measured by cardiac magnetic resonance (CMR) imaging [10]. This strong concordance reinforces its accuracy as a non-invasive surrogate for global RV function. We would like to reinforce that even though TAPSE and S′ are standard bedside metrics, they belong to a broader spectrum of diagnostic modalities used to quantify RV function in complex clinical scenarios.

Both pulsed-wave tissue Doppler and color-coded tissue Doppler techniques can be employed to derive S’ [11]. Regardless of the technique employed, S’ remains a highly reproducible parameter. A higher S’ velocity during systole correlates directly with superior RV systolic function. International consensus guidelines establish a normal reference value for S’ at ≥ 9.5 cm/s [12].

Parallel to RV dysfunction, venous congestion (VC) has emerged as a critical determinant of outcomes in HF. Elevated venous pressure, is a critical, often-overlooked aspect of hemodynamic stability. It can impair organ perfusion and lead to organ dysfunction. Assessment of specific veins using the Venous Excess Ultrasound (VExUS) score aids in the early detection of VC. This allows for better fluid management and prevents significant end-organ damage.

Right ventricular-pulmonary artery (RV–PA) coupling, assessed using Tricuspid annular plane systolic excursion (TAPSE)/Pulmonary artery systolic pressure (PASP) and S’/PASP ratios, has emerged as an important prognostic marker in heart failure patients. RV–PA uncoupling is associated with increased mortality, recurrent hospitalization, and adverse clinical outcomes, particularly in patients with reduced ejection fraction [13–15].

Currently, there is limited evidence examining the direct relationship between Doppler-derived RV systolic function (such as S′) and ultrasound-based congestion assessment using VExUS. Furthermore, it remains unclear whether combining these parameters can enhance bedside evaluation and risk stratification in patients with HF.

Therefore, our study aims to evaluate the relationship between RV systolic function (measured by S′) and systemic venous congestion (assessed by VExUS score) and explore whether an integrated ultrasound-based approach can improve bedside hemodynamic assessment in patients admitted with decompensated HF.

## Methods

### Ethics approval

This study was approved by the Narayana Health Academic Ethics Committee (Approval: # NHH/AEC-CL-2024-1249; September,2024). This trial was registered with Clinical Trial Registry-India (https://www.ctri.nic.in/), Trial Number - CTRI/2024/10/075219 on 14/10/2024. Informed consent was obtained from the patients before the screening process. Patients who declined consent were not included in the study.

### Reporting guideline adherence

This study was designed and reported as per the Strengthening the Reporting of Observational Studies in Epidemiology (STROBE) guidelines.

### Study objective and design

A prospective, single-centre, observational cohort study was conducted within the Cardiac Critical Care Unit (CCU), enrolling patients admitted with HF. This study was conducted in a 36 bedded CCU of a large tertiary care hospital located in Bengaluru, India, during a 6-month period (November 2024 - April 2025). Our CCU manages patients with HF, acute coronary syndrome, pre and post heart transplant and malignant arrhythmias. The primary objective of this study was to investigate the correlation between VC and S’ in HF patients. Secondary objective was to evaluate the prognostic implications of reduced S’ in the presence of VC.

After obtaining informed consent, a one-time comprehensive, multiparametric assessment of VC was conducted at admission within 24 h using an advanced echocardiographic approach. Particular emphasis was placed on the quantification of S′ using Tissue Doppler Imaging (TDI).

All participants underwent a standardized transthoracic echocardiographic examination in the supine position incorporating a structured VExUS protocol. This included assessment of inferior vena cava (IVC) diameter and respiratory collapsibility, along with pulsed-wave Doppler evaluation of hepatic, renal, and portal venous flow patterns. Based on these parameters, a composite VExUS score reflecting the severity of systemic VC was derived. Pulmonary congestion was assessed using a standardized Lung Ultrasound Score (LUS). Patients were grouped into different classes of HF based on the AHA classification based on the ejection fraction [16]. Further PASP measurements were also documented to calculate the RV-PA coupling based on both TAPSE/PASP and S’/PASP.

In addition, all participants were evaluated using the Get With The Guidelines (GWTG) score for clinical risk stratification, acute kidney injury (AKI) staging as per Kidney Disease: Improving Global Outcomes (KDIGO) criteria, and quantitative assessment of ascites as part of the comprehensive evaluation [17]. Central venous pressure (CVP), measured via central venous catheterization or estimated through detailed jugular venous pressure (JVP) assessment, was correlated with S′ values to provide an integrated evaluation of VC.

**Fig. 1:**
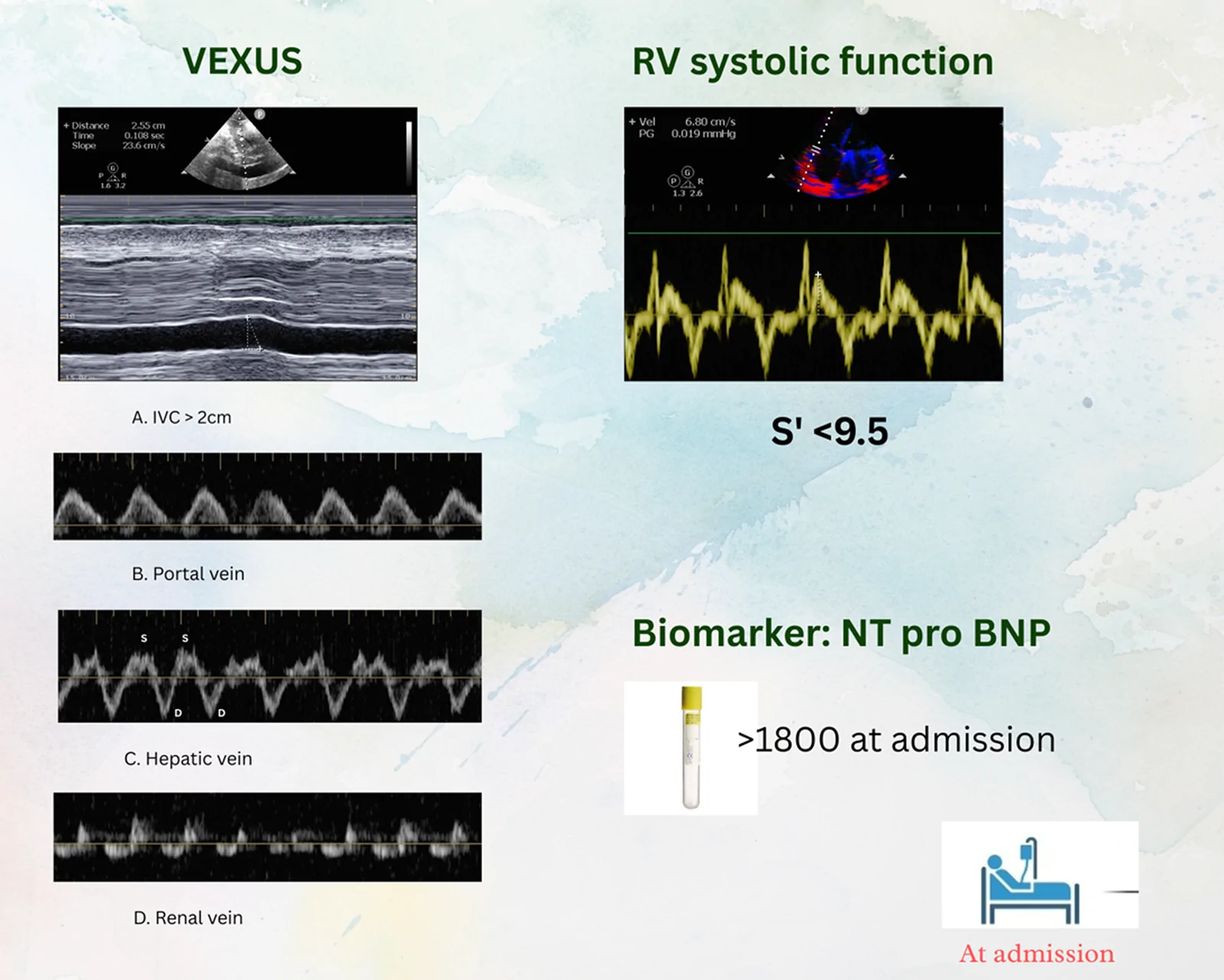
Comprehensive bedside assessment done at admission for a patient who presented with features of heart failure: The VExUS panel (left) includes: **A**: Inferior Vena Cava (IVC) ultrasound, showing a dilated IVC measuring >2 cm, indicative of increased right atrial pressure. **B**: Pulsed-wave Doppler of the portal vein, showed pulsatile waveform, with pulsatility index > 50% **C**: Pulsed-wave Doppler of the hepatic vein, showed S wave reversal. **D**: Pulsed-wave Doppler of the renal vein, illustrating a discontinuous monophasic flow with only diastolic phase. The RV Systolic Function panel (top right) shows a TDI trace with an S’ wave velocity < 9.5 cm/s, suggesting impaired right ventricular systolic function

All collected data were entered into a secure, de-identified electronic database. Statistical analyses were performed to explore correlations between VC parameters, S′, TAPSE, RV-PA coupling, RV functional reserve, renal function, fluid overload, and relevant clinical outcomes (see Fig. 1).

### Patient eligibility

**Fig. 2:**
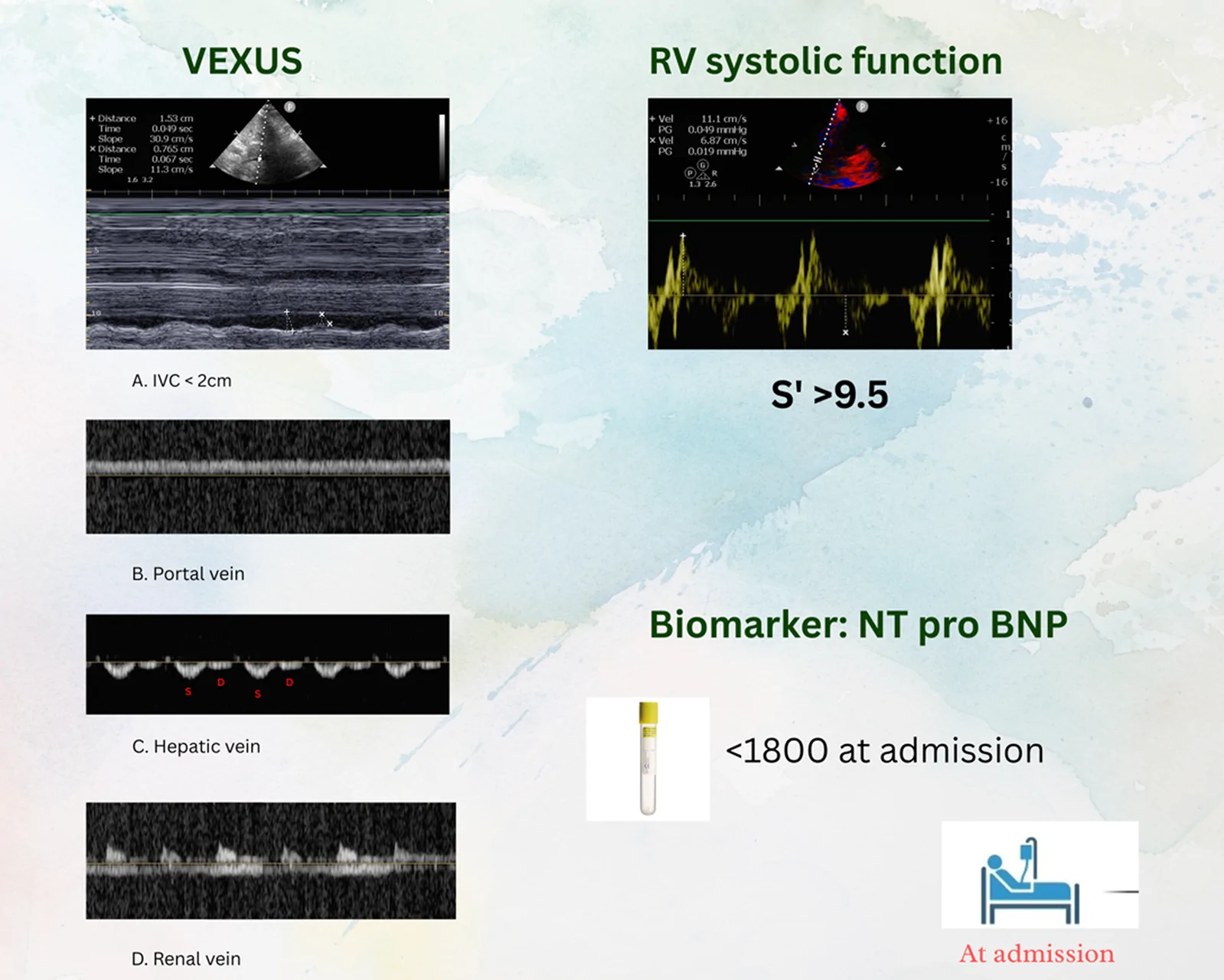
Comprehensive bedside assessment done at admission for a patient without features of venous congestion. The VExUS panel (left) includes: **A**: Inferior Vena Cava (IVC) ultrasound, showing a compressible IVC measuring <2 cm. **B**: Pulsed-wave Doppler of the portal vein, with pulsatility index < 30% **C**: Pulsed-wave Doppler of the hepatic vein, showing S > D pattern. **D**: Pulsed-wave Doppler of the renal vein, illustrating a continuous monophasic flow. The RV Systolic Function panel (top right) shows a TDI trace with an S’ wave velocity > 9.5 cm/s

Adult patients aged ≥ 18 years with HF diagnosed according to American Heart Association guidelines, undergoing clinically indicated echocardiography and able to provide informed consent were included. Patients in mechanical ventilator support with PEEP > 5 mmHg and Non-Invasive Ventilation (NIV) of Expiratory Positive Airway Pressure (EPAP) > 10 mmHg or with severe comorbidities such as end-stage renal disease on dialysis, advanced liver disease, malignancy with limited life expectancy, severe COPD, pregnancy, active systemic infections, or critical non-cardiac illnesses were excluded (see Fig. 2).

### Techniques for assessment

VExUS, S′, and LUS assessments were performed by a certified intensivist with over five years of expertise in critical care ultrasonography using a Philips CX50 ultrasound system. Doppler measurements were obtained during respiratory pauses at end-expiration to minimize artifacts [18]. S′ was measured at the lateral tricuspid annulus in the apical four-chamber view using TDI to capture peak systolic velocity accurately. RV systolic function was categorized based on an S′ velocity cutoff value of ≥ 9.5 cm/s. TAPSE cut off was taken as < 17 mm for defining RV failure [19]. As for S’/PASP, > 0.19 cm/s/mmHg was considered being coupled and ≤ 0.19 cm/s/mmHg as uncoupled (5). For TAPSE/PASP mm/mmHg, > 0.31 was taken as coupled and < 0.31 mm/mmHg is uncoupled [15]. LUS was performed using a linear probe across six lung zones bilaterally and scored using the standardized 36-point system [20].

### Sample size calculation

The sample size was calculated based on the proportion of association between renal congestion and poor RV function at a baseline of 67.6%, from the study by N. Bouabdallaoui et al. with a 95% Confidence Interval and 0.075 standard error [21]. It was calculated based on the following formula:

$${n}={z}1-\alpha22\times{P}(1-{P}){d}2$$

Here,

Significance level (1-𝛼) = 0.05.

Estimated Proportion (p) = 0.676.

Confidence level (Z) = 95%.

Estimated error (d) = 0.075.

Estimated Sample size (n) = 150 (Required Samples).

### Statistical analysis

Data were analysed using IBM SPSS software version 31. Continuous variables were expressed as mean ± standard deviation or median with interquartile range, while categorical variables were presented as frequency and percentage. Kruskal–Wallis test was used to compare S’ values and RV functional parameters across different VExUS grades. Categorical variables were compared using Chi-square test or Fisher’s exact test when expected cell counts were less than 5. Correlation between VExUS grade, S’, and RV functional parameters was assessed using Kendall’s Tau and Spearman correlation analyses as appropriate. To examine the relationship between BNP, S’, and VExUS grade, correlation analyses appropriate for skewed data (Pearson and Spearman), Kruskal–Wallis tests across VExUS grades, and multivariable log-linear regression modelling ln(BNP) as a function of S’ and VExUS grade were performed to evaluate their independent associations. Statistical significance was set at *p* < 0.05.

## Results

A total of 155 patients with HF were included in this study. The mean age of the cohort was 57.5 ± 14.51 years and 68% were male. The median GWTG HF risk score was 41 (IQR: 36–46), corresponding to an expected in-hospital mortality range of 1–5%. The observed in-hospital mortality rate was 10.3% (*n* = 16).

Hypertension was the most common comorbidity, affecting 71% (*n* = 110) of the patients, followed by diabetes mellitus (DM) in 58.7% (*n* = 91) and chronic kidney disease (CKD) in 12.3% (*n* = 19). The median LUS was 24 (IQR: 12–30), indicating moderate to severe pulmonary congestion across the cohort.

VC was graded using the VExUS scoring system, with 51% (*n* = 79) having no congestion, 8.4% (*n* = 13) with mild congestion, 13.5% (*n* = 21) with moderate congestion, and 27.1% (*n* = 42) presenting with severe congestion. RV systolic function was stratified using a threshold of ≥ 9.5 cm/s, with 65.2% (*n* = 101) and 34.8% (*n* = 54) below the threshold distinguishing preserved from impaired RV function, respectively.

Clinical signs of systemic congestion included raised jugular venous pressure in 69.7% (*n* = 108) of patients and ascites in 23.2% (*n* = 36). The mean CVP was 17.26 ± 7.41 mmHg. Regarding respiratory support, 78.7% (*n* = 122) were on spontaneous ventilation, 18.1% (*n* = 28) required NIV, and 3.2% (*n* = 5) were managed with invasive mechanical ventilation (Table 1).

**Table 1:** Patient, clinical characteristics at admission and outcome data

Parameter	*N* = 155 (100%)
Age	57.5 ± 14.51
Gender (Male)	106 (68.4)
GWTG Score	41 (36–46)
LUS	24 (12–30)
**Comorbid illness**	
DM	91 (58.7)
Hypertension	110 (71)
CKD	19 (12.3)
HF	
HFrEF	87 (56.1)
HFmrEF	29 (18.7)
HFpEF	39 (25.2)
**AKI Staging**	
0	117 (75.5)
1	11 (7.1)
2	21 (13.5)
3	6 (3.9)
**VExUS score**	
No congestion (Grade 0)	79 (51)
Mild (Grade 1)	13 (8.4)
Moderate (Grade 2)	21 (13.5)
Severe (Grade 3)	42 (27.1)
**RV S’ Category**	
≥ 9.5	101 (65.2)
< 9.5	54 (34.8)
TAPSE	15.87 ± 3.03
PASP	42.78 ± 18.95
Ascites	36 (23.2)
JVP Raised	108 (69.7)
CVP	17.26 ± 7.41
**Ventilation**	
Invasive Ventilation	5 (3.2)
NIV	28 (18.1)
SV	122 (78.7)
**Outcomes**	
In-Hospital Mortality	16 (10.3)
**AKI Stage at discharge**	
0	127 (81.9)
1	9 (5.8)
2	15 (9.7)
3	4 (2.6)

Patients with VExUS Grade 0, indicating no VC had a significantly higher proportion of preserved RV function (S′ ≥9.5 cm/s) compared to those with higher congestion grades. Specifically, 77.2% of VExUS grade 0 patients demonstrated preserved RV function, whereas only 52.6% of those with VExUS grades 1–3 met this criterion. Conversely, impaired RV function (S′ < 9.5 cm/s) was more prevalent in the higher VExUS grade group (47.4%) compared to the VExUS 0 group (22.8%) (Table 2).

**Table 2:** Association between VExUS score and RV S’ functional value

	VExUS 0 (*n* = 79)	VExUS Score 1–3 (*n* = 76)	P value
RV S’ ≥9.5	61 (77.2%)	40 (52.6%)	0.001*
RV S’ < 9.5	18 (22.8%)	36 (47.4%)	

Increasing VExUS grades were associated with progressive worsening of RV systolic function and RV–PA coupling parameters. In the overall cohort, TAPSE, TAPSE/PASP ratio, S’, and S’/PASP ratio showed significant reductions with increasing VExUS severity (*p* < 0.001 for all). Similar significant associations were observed in Heart failure with reduced ejection fraction (HFrEF) and Heart failure with preserved ejection fraction (HFpEF) patients, where worsening venous congestion correlated with lower TAPSE, S’, and RV–PA coupling indices. In Heart failure with mildly reduced ejection fraction (HFmrEF) patients, TAPSE and S’ did not show significant variation across VExUS grades probably attributable to low sample size; however, TAPSE/PASP and S’/PASP ratios were significantly reduced (*p* = 0.02), suggesting impaired RV–PA coupling despite relatively preserved conventional RV systolic parameters. Overall, higher VExUS grades were consistently associated with RV dysfunction and RV–PA uncoupling in heart failure patients across all groups (Table 3).

**Table 3:** Comparison of RV parameters based on VExUS groups

Group	Variable	VExUS 0	VExUS 1	VExUS 2	VExUS 3	*p*-value
Overall	TAPSE	17 (15, 19)	16 (15, 19)	15 (13, 17)	14 (12, 16)	< 0.001*
	TAPSE/PASP	0.60 (0.32, 0.78)	0.53 (0.30, 0.62)	0.38 (0.28, 0.54)	0.25 (0.19, 0.32)	< 0.001*
	S’	11.0 (10.0, 12.7)	12.6 (10.3, 15.0)	10.3 (8.0, 12.8)	8.6 (7.1, 11.5)	< 0.001*
	S’/PASP	0.36 (0.23, 0.47)	0.36 (0.26, 0.46)	0.27 (0.19, 0.41)	0.16 (0.12, 0.25)	< 0.001*
HFrEF	TAPSE	16 (15, 18)	16 (12, 16)	15 (13.5, 16.5)	13 (11.5, 16)	0.003*
	TAPSE/PASP	0.38 (0.29, 0.67)	0.30 (0.28, 0.57)	0.34 (0.26, 0.42)	0.27 (0.20, 0.34)	0.009*
	S’	10.8 (9.2, 11.8)	15.0 (7.5, 15.7)	10.3 (7.9, 12.4)	8.5 (7.0, 11.2)	0.04*
	S’/PASP	0.28 (0.20, 0.42)	0.27 (0.26, 0.36)	0.21 (0.19, 0.35)	0.17 (0.13, 0.26)	0.02*
HFmrEF	TAPSE	17 (16, 19)	19.5 (18, 20.3)	18 (15.8, 19)	12 (10.8, 14.5)	0.14
	TAPSE/PASP	0.68 (0.33, 0.79)	0.50 (0.46, 0.54)	0.58 (0.51, 0.66)	0.20 (0.19, 0.23)	0.02*
	S’	12.1 (10.2, 13.5)	13.1 (12.0, 13.6)	12.7 (11.0, 13.5)	9.7 (8.9, 10.8)	0.42
	S’/PASP	0.43 (0.32, 0.47)	0.35 (0.31, 0.39)	0.42 (0.35, 0.48)	0.17 (0.15, 0.19)	0.02*
HFpEF	TAPSE	19 (17, 20)	17.5 (14.3, 19)	14.5 (13.3, 15.8)	15 (14, 16)	< 0.001*
	TAPSE/PASP	0.78 (0.66, 0.82)	0.67 (0.51, 0.73)	0.50 (0.37, 0.62)	0.22 (0.19, 0.28)	< 0.001*
	S’	11.8 (11.0, 13.5)	12.0 (11.1, 15.0)	8.9 (8.4, 9.3)	8.2 (7.1, 11.5)	0.01*
	S’/PASP	0.46 (0.40, 0.58)	0.46 (0.39, 0.58)	0.32 (0.23, 0.41)	0.14 (0.08, 0.22)	< 0.001*

*p-value of < 0.05 was considered statistically significant

LUS were higher in patients with VExUS grade 1–3 compared with grade 0 (25.2 ± 9.4 vs 17.7 ± 10.4) and this difference was statistically significant (p < 0.001). When stratified by S’, those with S’≥9.5 had lower LUS than those with S’< 9.5 (19.5 ± 10.7 vs. 24.8 ± 9.4; *p* = 0.003), indicating greater ultrasound congestion at lower S’ values. Baseline AKI stage distribution did not differ significantly between VExUS strata (overall *p* = 0.07) or between S’ categories (overall *p* = 0.71), with most patients in AKI Stage 0 across groups (Table 4).

**Table 4:** Association of LUS and AKI stages with VExUS score and S’

	VExUS grade 0 (*n* = 79)	VExUS grade 1 to 3 (*n* = 76)	*P* value	S’ ≥9.5 (*n* = 101)	S’ <9.5 (*n* = 54)	*P* value
LUS	17.7 ± 10.4	25.2 ± 9.4	< 0.001	19.5 ± 10.7	24.8 ± 9.4	0.003*
**AKI Baseline**						
AKI Stage 0	66 (83.5%)	51 (67.1%)	0.07	74 (73.3%)	43 (79.6%)	0.71
AKI Stage 1	5 (6.3%)	6 (7.9%)		8 (7.9%)	3 (5.6%)	
AKI Stage 2	7 (8.9%)	14 (18.4%)		14 (13.9%)	7 (13%)	
AKI Stage 3	1 (1.3%)	5 (6.6%)		5 (5%)	1 (1.9%)	

Exploratory analysis demonstrated a significant inverse correlation between increasing VExUS grades and RV functional as well as RV–PA coupling parameters, particularly in HFrEF and HFpEF patients. Patients with higher VExUS grades more frequently exhibited impaired TAPSE, S′, TAPSE/PASP, and S′/PASP values, further supporting the association between worsening VC and RV dysfunction (Tables S1-S2). LUS demonstrated significant correlations with VExUS grade and RV functional parameters, with worsening pulmonary congestion associated with lower TAPSE, S′, TAPSE/PASP, and S′/PASP values. In contrast, baseline AKI stage did not show a significant correlation with either RV functional indices or VExUS grade, suggesting a weaker relationship between renal dysfunction and single time-point congestion assessment in this cohort (Tables S3-S24).

## Discussion

The aim of our study was to specifically evaluate the correlation between S′ and VExUS grades, a relationship that has not been extensively explored in previous literature. We sought to assess VC in patients with HF using both VExUS and S′, and to determine whether higher VExUS grades corresponded to lower S′ values. The objective was to explore whether S′ could serve as a practical guide to VC, compared to conventional measurements such as TAPSE, thereby functioning as a simple bedside tool in the assessment and management of HF.

Statistical analysis demonstrated a significant inverse correlation between VExUS grade and S′, as shown in Table 2, confirming a consistent trend in which worsening VC was associated with declining RV contractile function. This finding indicates an inverse relationship between the degree of systemic VC and RV systolic performance. Our findings are consistent with previous studies demonstrating that worsening venous congestion and elevated right-sided filling pressures are associated with impaired RV function, RV–PA uncoupling, and poorer clinical outcomes in HF patients [22]. The present study further extends these observations by specifically demonstrating a significant inverse relationship between VExUS grading and S′, supporting the utility of S′ as a simple bedside marker of venous congestion and RV dysfunction.

These results support the pathophysiological link between worsening VC and RV Failure. Higher VExUS grades reflect increasing systemic VC, which appears to parallel deterioration in RV contractility, potentially due to increased pulmonary afterload, elevated RV filling pressures, and impaired forward flow. This association highlights the concept of RV–venous coupling, whereby sustained preload elevation and afterload mismatch contribute to RV decompensation.

Importantly, incorporating a Doppler-derived RV functional parameter alongside VExUS may improve the diagnostic accuracy and clinical utility of bedside ultrasound in guiding decongestive therapy. The combined assessment allows for more nuanced identification of patients at risk for further hemodynamic compromise than either metric alone. As a quantitative and load-sensitive parameter, S′ may provide early insight into RV dysfunction before overt clinical deterioration becomes evident.

Increasing VExUS grades were associated with progressive worsening of RV systolic function and RV–PA coupling parameters. In the overall cohort, TAPSE, TAPSE/PASP ratio, S′, and S′/PASP ratio showed significant reductions with increasing VExUS severity (*p* < 0.001 for all). Similar significant associations were observed in HF Type 1 and HF Type 3 patients, where worsening venous congestion correlated with lower TAPSE, S′, and RV–PA coupling indices. In HF Type 2 patients, TAPSE and S′ did not show significant variation across VExUS grades; however, TAPSE/PASP and S′/PASP ratios were significantly reduced (*p* = 0.02), suggesting impaired RV–PA coupling despite relatively preserved conventional RV systolic parameters. In addition to conventional RV systolic indices, the S′/PASP ratio demonstrated a consistent decline with worsening VExUS grades, supporting its potential role as a marker of RV–PA uncoupling. As S′ is simple, rapid, and less angle-dependent in routine echocardiography, S′/PASP may represent a practical bedside parameter for evaluating the interaction between RV contractility and pulmonary vascular load in HF patients. These findings suggest worsening RV systolic performance and impaired RV–PA coupling in patients with severe venous congestion.

LUS was compared across groups stratified by VExUS grade and S′. Patients with VExUS Grade 0 had a significantly lower mean LUS (17.7 ± 10.4) compared to those with VExUS Grades 1–3 (25.2 ± 9.4; *p* < 0.001), indicating a clear association between systemic VC and pulmonary fluid overload. Similarly, patients with preserved RV function demonstrated a lower mean LUS (19.5 ± 10.7) than those with impaired RV function (24.8 ± 9.4; *p* = 0.003). These findings suggest that both higher VExUS grades and reduced S′ velocities are associated with more severe pulmonary congestion.

In contrast, AKI assessed on admission did not show a significant correlation with either S′ or VC as quantified by VExUS. This lack of association is consistent with prior evidence indicating that single time-point measurements may not adequately capture the dynamic cardiorenal interactions involved in AKI development. Although patients with higher VExUS grades exhibited numerically higher stages of AKI, the association did not achieve statistical significance.

The prognostic utility of S′ extends beyond its role in RV functional assessment. Incorporating S′ into the evaluation of cardio-renal syndrome may provide deeper insights into the interaction between cardiac and renal dysfunction, potentially enabling more targeted therapeutic strategies and improved patient outcomes. Additionally, the role of S′ in guiding fluid management underscores its versatility in routine clinical practice. The combined use of VExUS and RV functional assessment may also help identify patients who could benefit from more aggressive or tailored decongestive strategies. Serial monitoring of these parameters during hospitalization may provide additional insight into treatment response and evolving hemodynamic status.

Furthermore, BNP demonstrated a significant inverse association with S′ and a positive association with VExUS grade in the attached dataset. BNP levels decreased with increasing S′ values based on both Pearson and Spearman correlation analyses, while higher VExUS grades were associated with increased BNP levels, as shown by positive rank-based correlations and significant distributional differences across grades. When modeled together in a log-linear regression of ln(BNP), both S′ and VExUS grade remained independently significant, indicating that congestion severity and RV systolic function exert independent effects on BNP levels.

Beyond the primary objectives, AKI and mortality were also evaluated. Neither outcome demonstrated statistical significance, possibly due to limited sample size, relatively low event rates, and the multifactorial mechanisms contributing to AKI and mortality in HF patients. Although patients with higher VExUS grades exhibited a numerically greater incidence of in-hospital mortality, this difference did not reach statistical significance. Similarly, patients with impaired S′ appeared more frequently among non-survivors, but this trend was also not statistically significant. These findings highlight the multifactorial nature of HF outcomes, where no single parameter adequately predicts early in-hospital mortality.

Overall, our study demonstrates a strong inverse relationship between the severity of VC, as quantified by VExUS, and RV systolic function measured by S′. These findings suggest that S′ may serve as a valuable and easily accessible bedside marker for assessing VC in patients with HF. In resource-limited settings where invasive hemodynamic monitoring is unavailable, non-invasive tools such as S′ and VExUS offer practical alternatives for congestion assessment. Overall, our findings suggest that integration of VExUS grading with RV systolic and RV–PA coupling assessment may provide a comprehensive, non-invasive bedside approach for evaluating congestion severity and hemodynamic compromise in HF patients. Further multicenter studies with larger cohorts and longitudinal follow-up are warranted to validate the prognostic utility of S′ and S′/PASP in this population.

### Limitations

All assessments were performed within 24 h of admission, limiting evaluation of longitudinal changes and treatment responsiveness. AKI classification was based on admission criteria without standardized serial reassessment. The absence of significant associations with mortality and AKI is likely related to the limited sample size. Larger prospective studies with serial measurements are required to validate the combined prognostic utility of VExUS and S′ in HF populations. Patients with pulmonary hypertension were not specifically stratified in this study because as majority of our patients did not have a pulmonary artery catheter. We have not assessed the GLS in our routine echocardiographic examination and hence do not have the data on FAC and RV free wall strain.

## Conclusion

Our findings highlight the value of combining VExUS and S’ with LUS, as both high VExUS grades and reduced S’ values were independently associated with more severe pulmonary congestion. Ultimately, our results support an integrated, multi-modal ultrasound approach to assess and manage HF, using S’ and VExUS to guide decongestive therapy and identify patients at risk for hemodynamic decompensation.

## Supplementary Information

Below is the link to the electronic supplementary material.

Supplementary Material 1

## Data Availability

The datasets used and/or analysed during the current study are available from the corresponding author on reasonable request.

## Abbreviations

HF: Heart Failure
RV: Right ventricle
VC: Venous Congestion
IVC: Inferior Vena Cava
AKI: Acute Kidney Injury
S': Tricuspid annular systolic velocity
CCU: Cardiac Critical care Unit
VExUS: Venous Excess Ultrasound
LUS: Lung Ultrasound Score
