# Supplementary material for: Utilizing bedside VExUS and the S-prime velocity for real-time assessment of venous congestion on right ventricular function in heart failure
Source: Egypt Heart J. 2026 Oct 6;78:76. doi: 10.1186/s43044-026-00788-7 (PMC13642859; doi:10.1186/s43044-026-00788-7)
Supplement: Supplementary file 1 [file 43044_2026_788_MOESM1_ESM.pdf]

Codes

HF type	1. HFrEF<br>2. HFmrEF<br>3. HFpEF
TAPSE	0 ≤17<br>1 > 17
TAPSE/ PASP	0 < 0.31<br>1 > 0.31
S’	0 ≤ 9.5<br>1 > 9.5
S’/ PASP	0 < 0.19<br>1 > 0.19

Table-S1 Correlation of VExUS grade with RV function based on ejection fraction failure

Group	Variable	Correlation (95% CI)	p-value
**Overall**	S’	-0.29 (-0.44 to -0.14)	< 0.001*
	S’/PASP	-0.46 (-0.58 to -0.33)	< 0.001*
	TAPSE	-0.45 (-0.56 to -0.32)	< 0.001*
	TAPSE/PASP	-0.49 (-0.61 to -0.36)	< 0.001*
**HFrEF**	S’	-0.23 (-0.44 to -0.02)	0.03*
	S’/PASP	-0.30 (-0.49 to -0.09)	0.004*
	TAPSE	-0.40 (-0.57 to -0.21)	< 0.001*
	TAPSE/PASP	-0.36 (-0.54 to -0.15)	< 0.001*
**HFmrEF**	S’	-0.14 (-0.49 to 0.25)	0.46

	S’/PASP	-0.44 (-0.72 to -0.03)	0.01*
	TAPSE	-0.13 (-0.52 to 0.34)	0.51
	TAPSE/PASP	-0.42 (-0.73 to 0.00)	0.02*
**HFpEF**	S’	-0.48 (-0.71 to -0.19)	0.002*
	S’/PASP	-0.68 (-0.81 to -0.48)	< 0.001*
	TAPSE	-0.72 (-0.83 to -0.56)	< 0.001*
	TAPSE/PASP	-0.67 (-0.84 to -0.42)	< 0.001*

*p-value of <0.05 was considered statistically significant (Spearman correlation test)

Table-S2 Association between VExUS grade and RV function

Group	Variable	Level	VExUS 0	VExUS 1–3	p-value
Overall	TAPSE	0	34 (43.0%)	57 (75.0%)	< 0.001*
		1	45 (57.0%)	19 (25.0%)	
	TAPSE/PASP	0	19 (24.1%)	40 (52.6%)	< 0.001*
		1	60 (75.9%)	36 (47.4%)	
	S’	0	18 (22.8%)	36 (47.4%)	< 0.001*
		1	61 (77.2%)	40 (52.6%)	
	S’/PASP	0	11 (13.9%)	31 (40.8%)	< 0.001*
		1	68 (86.1%)	45 (59.2%)	
HFrEF	TAPSE	0	27 (61.4%)	36 (83.7%)	0.03
		1	17 (38.6%)	7 (16.3%)	
	TAPSE/PASP	0	15 (34.1%)	22 (51.2%)	0.16
		1	29 (65.9%)	21 (48.8%)	
	S’	0	12 (27.3%)	22 (51.2%)	0.03*
		1	32 (72.7%)	21 (48.8%)	

	S'/PASP	0	9 (20.5%)	17 (39.5%)	0.08
		1	35 (79.5%)	26 (60.5%)	
**HFmrEF**	TAPSE	0	5 (29.4%)	5 (41.7%)	0.77
		1	12 (70.6%)	7 (58.3%)	
	TAPSE/PASP	0	3 (17.6%)	3 (25.0%)	0.66
		1	14 (82.4%)	9 (75.0%)	
	S'	0	4 (23.5%)	3 (25.0%)	1
		1	13 (76.5%)	9 (75.0%)	
	S'/PASP	0	1 (5.9%)	3 (25.0%)	0.27
		1	16 (94.1%)	9 (75.0%)	
**HFpEF**	TAPSE	0	2 (11.1%)	16 (76.2%)	< 0.001*
		1	16 (88.9%)	5 (23.8%)	
	TAPSE/PASP	0	1 (5.6%)	15 (71.4%)	< 0.001*
		1	17 (94.4%)	6 (28.6%)	
	S'	0	2 (11.1%)	11 (52.4%)	0.008*

		1	16 (88.9%)	10 (47.6%)	
	S'/PASP	0	1 (5.6%)	11 (52.4%)	0.001*
		1	17 (94.4%)	10 (47.6%)	

*p-value of <0.05 was considered statistically significant (Chi square test/ Fischer exact test if count < 5)

Table-S3 Correlation between RV function and VExUS grade with LUS

**Variable**	**Correlation (95% CI)**	**p-value**
TAPSE	-0.29 (-0.43, -0.13)	<0.001*
TAPSE/PASP	-0.40 (-0.53, -0.26)	<0.001*
S’	-0.17 (-0.31, -0.02)	0.04
S’/PASP	-0.38 (-0.51, -0.24)	<0.001*
VExUS grade	0.39 (0.26, 0.53)	<0.001*

*p-value of <0.05 was considered statistically significant

Table-S4 Correlation between baseline AKI stage and VExUS grade with LUS

Variable	Correlation (95% CI)	p-value
TAPSE	-0.01 (-0.16, 0.14)	0.9
TAPSE/PASP	0.08 (-0.08, 0.22)	0.34
S’	-0.02 (-0.17, 0.13)	0.85
S’/PASP	0.03 (-0.12, 0.17)	0.68
VExUS grade	0.07 (-0.08, 0.23)	0.36

*p-value of <0.05 was considered statistically significant
